# Rapid and Label-Free Analysis of Antigen–Antibody Dynamic Binding of Tumor Markers Using Piezoelectric Quartz Crystal Biosensor

**DOI:** 10.3390/bios13100917

**Published:** 2023-10-07

**Authors:** Yan Chen, Huashan Shi

**Affiliations:** 1 School of Information Engineering, Southwest University of Science and Technology, Mianyang 621010, China; 2 Department of Biological Therapy, West China Hospital, Sichuan University, Chengdu 610047, China; shihuashan@scu.edu.cn

**Keywords:** piezoelectric (PZ) quartz crystal biosensor, binding kinetics, molecular diagnostics, cancer, clinical serum samples

## Abstract

Quantitative biomacromolecular diagnosis is rapidly developing in molecular oncology. In this study, we developed a continuous flow immunoassay device based on a piezoelectric (PZ) quartz crystal biosensor fabricated with whole-electrode occupation for the quantitative molecular diagnosis of tumor markers such as alpha-fetoprotein (AFP). Only one face of the crystal was in contact with the serum sample during the assays. First, the characteristics of AFP and anti-AFP binding kinetics, such as the optimal time for immune response, the average antigen binding rate, the kinetic constants and the optimal standard curve, were investigated. The overall immunoreaction time was only 12 min, the average antigen binding rate of AFP was 45.9 ng/min, the concentration range of AFP detection was 18.8–1100 ng/mL and the association rate constant ($k_{\mathrm{on}}$), dissociation rate constant ($k_{\mathrm{off}}$) and equilibrium dissociation constant (K_D_) were $5.58\times 10^{4}{\;M}^{-1}s^{-1}$,$1.79\times 10^{-5}{\;s}^{-1}$ and $3.21\times 10^{-10}\;M$, respectively. This sensing system was further validated by detecting AFP values from clinical serum samples, which were obtained from pregnant women, liver and lung cancer patients and those undergoing liver cancer screening. No cross-reactivity with lung cancer markers were found, and the detection results were in good agreement with the radioimmunoassay (RIA) results, with a relative deviation of no more than 3.7% and correlation coefficient r of 0.9998. Therefore, the developed immunoassay device has the potential to be used in large-scale screening for cancers, as well as in novel high-affinity binding drug development.

## 1. Introduction

Cancer is one of the major modern health problems, ranking first among all the causes of death. Before the initial cancer cells convert into a malignant entity and when the malignant entity is small, patients generally do not perceive the symptoms of cancer. Generally, when patients go to the hospital with symptoms that are indicative of cancer, most cancer patients are at an advanced stage [1], obtaining a poor 5-year survival rate. An immunoassay (IA) is a biochemical test that detects the presence or concentration of a defined analyte in a specimen by using a specific antibody. Tumor marker diagnostic tests are immunoassays that measure cancer risk and indicate the presence of tumors in the tissue. Nowadays, although the sensitivity and specificity of the routine laboratory detection of tumor markers are not 100%, it plays a vital role in screening diseases and is currently one of the most important ways to detect asymptomatic micro-focal tumors in the early stage. The accurate detection of tumor markers requires a broad understanding of antigen–antibody binding dynamics. Many popular analytical approaches, such as enzyme-linked immunosorbent assays (ELISA) and chemiluminescence immunoassays (CLIA), have the significant disadvantage of requiring labeling. The most common label-free method currently used is surface plasmon resonance (SPR). However, it suffers from disadvantages such as non-specific adsorption and temperature sensitivity [2,3]. Several other label-free methods, such as backscattering interferometry (BSI) [4,5], bio-layer interferometry (BLI) [6] and isothermal titration calorimetry (ITC) [7,8,9], have received strong support from researchers. Nevertheless, they suffer from several shortcomings, including the need for knowledge about the refractive index, a lack of sensitivity, and a time-consuming nature. Therefore, it is of considerable interest to research label-free, specific, stable, rapid, sensitive, simple and real-time alternative methodologies for the characterization of the antigen–antibody binding reaction kinetics of tumor markers.

A piezoelectric (PZ) quartz crystal biosensor is a novel type of biosensor that perfectly combines the properties of quartz crystal, including piezoelectric, zero-temperature coefficient cuts and a high Q-factor with the characteristic of the high specific recognition of an immune response. It is an ultra-sensitive mass-sensing device that is also called a quartz crystal microbalance (QCM) and can be used to study antigen–antibody binding dynamics in tumor markers. In PZ-sensing technology, the antibody is usually immobilized onto the electrode of the quartz crystal. The adsorption of the antigen onto the crystal with the coated antibody can be identified by measuring the corresponding change in the resonant frequency of the quartz resonator. This is the well-known Sauerbrey effect [10,11], as illustrated in Equation (1).

(1) $$\Delta f=-\frac{2f_{0}{}^{2}}{A{\left({\bar{C}}_{66}\rho_{q}\right)}^{\frac{1}{2}}}\Delta m$$

where $f_{0}$ is the fundamental resonant frequency (in Hz), Δm is the mass change in the crystal surface (in g), Δf is the measured frequency shift (in Hz), A is the area of the electrode surface (in cm^2^), ${\bar{C}}_{66}=2.947\times 10^{11}\;g/\mathrm{cm}\cdot {\;s}^{2}$ is the shear modulus of the quartz, and $\rho_{q}=2.648{\;g/\mathrm{cm}}^{3}$ is the density of the quartz. In recent years, although PZ-sensing technology has been applied in the field of bioanalytics with the characteristics of being label-free, simple, highly sensitive and highly specific [12,13,14,15], it has not been applied in clinical practice until now due to the lack of ideal analysis time and the lack of research on the binding kinetics of antigens and antibodies.

In recent years, according to the statistics [16,17], the mortality rate of liver cancer has been increasing. Fortunately, for early-stage liver cancer, the 5-year survival rate is improved significantly after systematic and standardized treatment. Thus, early-stage liver cancer detection is of great significance for successful treatment, and in this study, we mainly studied the quantitative molecular diagnosis of alpha-fetoprotein (AFP).

The aim of this study was to explore the antigen–antibody binding kinetics of a tumor marker using PZ-sensing technology to achieve quantitative molecular diagnosis. This exploration included three aims: (a) The first was to develop a continuous flow cell measurement system for a PZ biosensor to improve the kinetics of the reaction and increase the concentration of antigen–antibody complexes. One advantage of this procedure and the device developed is that they do not employ any labeled species, thus simplifying the operation enormously. (b) The second was to investigate the binding kinetics of AFP and anti-AFP. (c) The third was to validate this continuous flow cell-sensing system by determining the AFP values of clinical serum samples. First, a continuous flow immunoassay device based on a PZ immunosensor fabricated with whole-electrode occupation was designed. Then, the characteristics of AFP and anti-AFP binding kinetics, such as the optimal time for an immune reaction, the average antigen binding rate, kinetic constants and the optimal standard curve, were investigated. Particular attention was given to the practicability of the AFP-level analysis of clinical sera using this PZ-sensing system, including its stability, specificity, accuracy, precision and regeneration.

## 2. Materials and Methods

### 2.1. Reagents

Recombinant staphylococcus aureus protein A (SPA) and bovine serum albumin (BSA) were obtained from Sigma Co. (Livonia, MI, USA). Mouse anti-AFP monoclonal antibody was purchased from Shanghai Linc-Bio Science Co., Ltd. (Shanghai, China). AFP standard antigen substance and clinical serum samples were provided by Sichuan Science City Hospital (Mianyang, China). Phosphate-buffered saline (PBS, pH 7.4) was composed of 2 mM KH_2_PO_4_, 8 mM Na_2_HPO_4_, 136 mM NaCl and 2.6 mM KCl. All other reagents were of analytical grade.

### 2.2. Apparatus

Customized quartz crystals were produced by Chengdu Spaceon Electronics Co., Ltd. (Chengdu, China) for this study, with the aim of maintaining the mechanical strength of the crystals and obtaining a high mass sensitivity [18]. Each of these special quartz crystals (AT-cut with a $35{}^{\circ}10^{\prime }$ angle, 8 mm in crystal diameter, 0.185 mm in thickness, 6 mm in electrode diameter and 10 MHz 3rd overtone) was subjected to its 3rd overtone resonance frequency using a homemade Pierce-Gate crystal oscillator circuit. Resonator frequency measurements were taken using Agilent universal frequency counter 53131A (Palo Alto, CA, USA), with a resolution of 0.1 Hz. The thermostat WB-350 was obtained from Wiggens Technology Co., Ltd. (Beijing, China) to control the experimental temperature.

### 2.3. PZ Biosensor Surface Modification

Prior to modification, two important pre-treatments had to be performed on each of the quartz crystals. First of all, the intersection between the electrode and the electrical connection was sealed with silastic film in order to avoid it being corroded by the chemical substances in the assay. Otherwise, conductivity would have been lost, resulting in the resonator failing to work. Next, each of the crystal had to be thoroughly cleaned to eliminate any possible contaminants. The cleaning process was as follows: the crystals were immersed in 1 M NaOH and 1 M HCl, respectively, for 15 min, and then, washed with ethanol (95%) and deionized water three times and blown dry with nitrogen steam. Then, a hydrophilic gold surface was obtained. The third harmonic frequency (f_1_) of the unloaded PZ sensor was recorded in the gas phase.

The pre-treated crystals were prepared for AFP antibody immobilization. First, 5 μL of the 10 mg/mL SPA solution in the PBS was slowly injected onto the contact electrode surface via flow control and incubated for 20 min at 22 °C. Before the frequency f_2_ was tested, the crystal was washed with PBS and distilled water three times, and it naturally air-dried. Thereafter, the crystal was immersed in 2 μL 8 mg/mL AFP monoclonal antibody solution to be incubated for 2 h at 22 °C, followed by a thorough rise with PBS and distilled water, and it dried at room temperature. The frequency of the AFP antibody film-loading PZ sensor was recorded as f_3_. Finally, 5 μL of the 5% BSA was applied onto the antibody-coated electrode surface and incubated for 1 h at 25 °C to block the nonspecific binding sites. In order to eliminate the damping loading effect induced by the liquid, another crystal called the reference crystal was required for each assay that did not undergo anti-AFP monoclonal antibody modification but was coated with SPA and blocked with BSA. These modified crystals were then stored at 4 °C in a humid environment.

### 2.4. Measurement Procedures

Before the measurement was carried out, each of the stored crystals was removed from the refrigerator and installed into the designed test system using electrode connection wires. Ten minutes after setting the operating temperature to 25 °C, the initial resonance frequency was determined. Then, each of the 1 mL PBS solutions containing different concentrations of AFP standard antigen was introduced into the contact well via the flow control of the liquid. The immunoreaction was immediately transformed into a frequency change in the crystal resonator, and the frequencies were recorded in real time, online, every 1 min after the frequency changed until equilibrium was reached. The same measurement procedure was carried out on the reference crystal. After the frequency shift ($\Delta F_{r}$) of the reference crystal was subtracted from the working crystal data ($\Delta F_{w}$), the frequency changes in the immunoreaction (ΔF) were obtained. Finally, clinical serum samples were measured in the same way. The flow rate of the liquid was 100 μL/min throughout the assay.

## 3. Results and Discussion

### 3.1. Measurement System of PZ Biosensor

**Figure 1:**
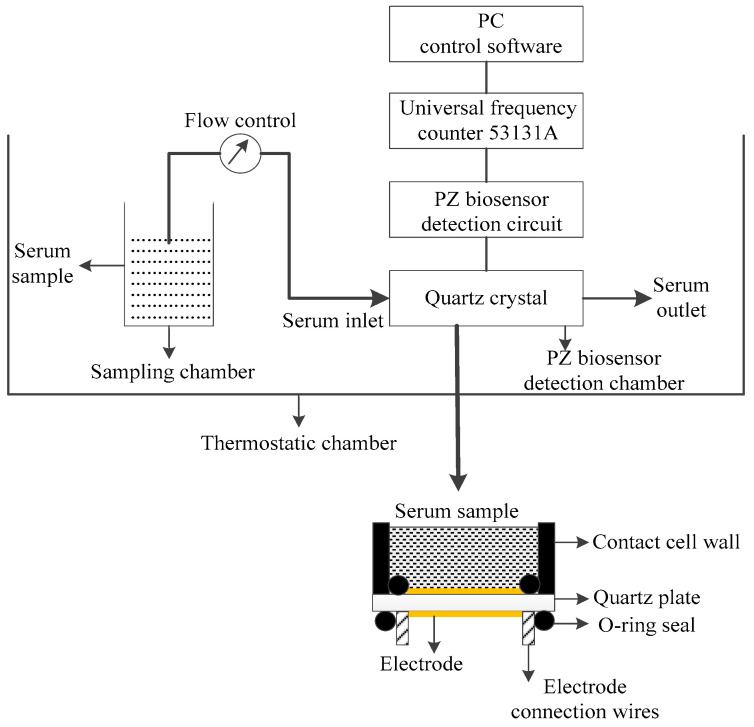
PZ biosensor measurement system for the kinetics of antigen–antibody binding of tumor markers.

The PZ biosensor test system is a core PZ-sensing technology. The establishment of a quartz crystal solution phase-sensing system should be considered from three perspectives. First, the mode of crystal contact with the serum needs to be determined. In PZ immunoassays, specific recognition between the antigen in a serum and the immobilized antibody on the crystal surface can be divided into two steps [19,20]. The first step is an antigen transport process, which is termed mass transport, and the second step is an antigen–antibody binding process. Whether the detection is performed via drip or immersion, antigen–antibody binding on the crystal is faster than mass transport is. As a result, an antigen-free region, which is called the depletion layer, is generated on the electrode surface. This condition can lead to a longer immune response time and limit the sensitivity of the PZ biosensor. Therefore, we employed the idea of microfluidics technology [21,22] to dynamically inject the serum, thereby speeding up the mass transfer process, ultimately shortening the immune response time and improving the mass sensitivity of the PZ biosensor. Second, the width of the crystal area in contact with the serum needs to be chosen carefully. The concentration of antigen–antibody complexes strongly depends on the width of the crystal surface subjected to the reaction [23]. Because it increases as the width of the surface reaction increases, the reaction surface is designed to occupy the whole of the electrode surface. Third, if both quartz crystal electrodes are immersed in the liquid, the vibration of the resonator can easily cease due to the anelastic properties of the solution film. Therefore, a flow cell must be designed to make sure that the solution is in contact with only one crystal surface in order to make the quartz crystal resonator reach a stable oscillation. The whole measurement test system is shown in Figure 1.

### 3.2. Oscillation Study of Quartz Crystal Resonator

A piezoelectric quartz crystal was placed and sealed in a homemade cell with two O-ring seals, and the O-ring was placed on both sides of the electrode to ensure that the whole electrode was in contact with the liquid. Only one face of the electrode was allowed to be in contact with the serum during the assays. Due to the energy trapping effect of the electrode, this clamping method can ensure that the resonator still has a high Q value after installation. Under the same experimental conditions, the resonant frequencies of different quartz crystal resonators were monitored, determining that the quartz crystal resonator has a stable frequency with a stability of ±1 Hz in liquid phase sensing.

### 3.3. Optimal Immune Response Time Selection

**Figure 2:**
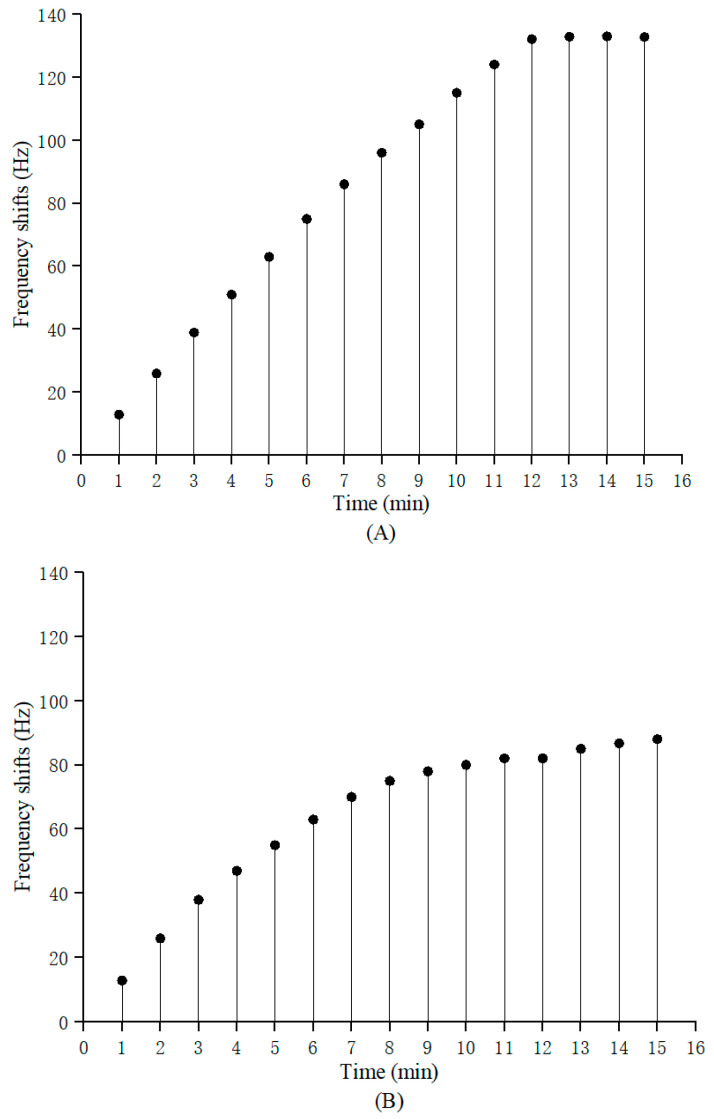
Selection of optimal response time of PZ biosensor. (**A**) Relationship between measurement time and frequency response of continuous flow detection. (**B**) Relationship between measurement time and frequency response as per static detection.

In immunoassay analysis, in addition to having a high detection accuracy, the detection time, which strongly depends on the time of the antigen–antibody immune response, is also expected to be as short as possible. While increasing the reaction temperature can increase the chance of collision between the antigen and antibody molecules and accelerate the formation of an antigen–antibody complex, an overly high temperature will deactivate the antigen or antibody and affect the experimental results. Most importantly, the frequency of the quartz crystal resonator is strongly temperature-dependent [24], and an overly high or low temperature will cause large frequency deviations in the test results. Fortunately, the AT-cut crystal at the $35{}^{\circ}10^{\prime }$ angle used in our investigation has a zero frequency temperature coefficient near 25 °C [25]. Therefore, the temperature of the immune response was set at 25 °C, and a 500 ng/mL AFP standard substance was continuously released onto the crystal at a 100 μL/min flow rate. The frequencies were recorded in real time every 1 min, and the monitoring duration was 15 min. When the maximum values in the frequency were acquired, this was the optimal time for the immune response, as shown in Figure 2. It can clearly be observed from Figure 2A that the frequency shifts of the sensor increased with increasing immune reaction times within 12 min; between 12 and 15 min, the resonant frequency of the sensor hardly changed, indicating that 12 min was the best immune response time in this study.

The static liquid detection time was also investigated, as shown in Figure 2B, and a response comparison between the two injection methods was made, as shown in Table 1 and Table 2, respectively. In order to eliminate the non-mass effect of the sensor, which was induced via the damping loading of the antigen solution, the frequency shifts of the reference crystal ($\Delta F_{r}$) and the working crystal ($\Delta F_{w}$) were measured during each detection. $\Delta F_{r}$ was subtracted from $\Delta F_{w}$ in the final analysis. Then, the absolute analyte mass immobilized on the senor was estimated using Equation (1). Obviously, a continuous flow with a high flow rate was very beneficial; it reduced sample dispersion, improved the mass transport, ensured that the rate of antigen binding did not sharply decay with the immune time and, finally, shortened the detection time.

### 3.4. Determination of Kinetic Constants

The precise evaluation of antibody binding kinetics plays a vital role in the accuracy and repeatability of immunoassays. Kinetics determines whether the antigen–antibody complex forms or dissociates within a given time span. In this investigation, 8 mg/mL AFP antibody and 1 mL AFP sample in a concentration range between 100 ng/mL and 600 ng/mL were used. Each sample injection was carried out at t = 0, and the antigens and antibodies fully reacted for 15 min. The dissociation time was extended to 30 min. After each sample measurement, the crystal surface was regenerated via a 5 min injection of Piranha solution and 2 min of washing with distilled water. To determinate the association rate constant ($k_{\mathrm{on}}$) and dissociation rate constant ($k_{\mathrm{off}}$), the 1:1 Langmuir binding model was used to fit the experimental data, and global curve fitting analysis was performed [26]. $k_{\mathrm{on}}$ and $k_{\mathrm{off}}$ were experimentally determined and were used to calculate the equilibrium dissociation constant (K_D_) as follows:

(2) $$K_{D}=\frac{k_{\mathrm{off}}}{k_{\mathrm{on}}}$$

These equilibrium constants are listed in Table 3.

### 3.5. Optimal Standard Curve

**Figure 3:**
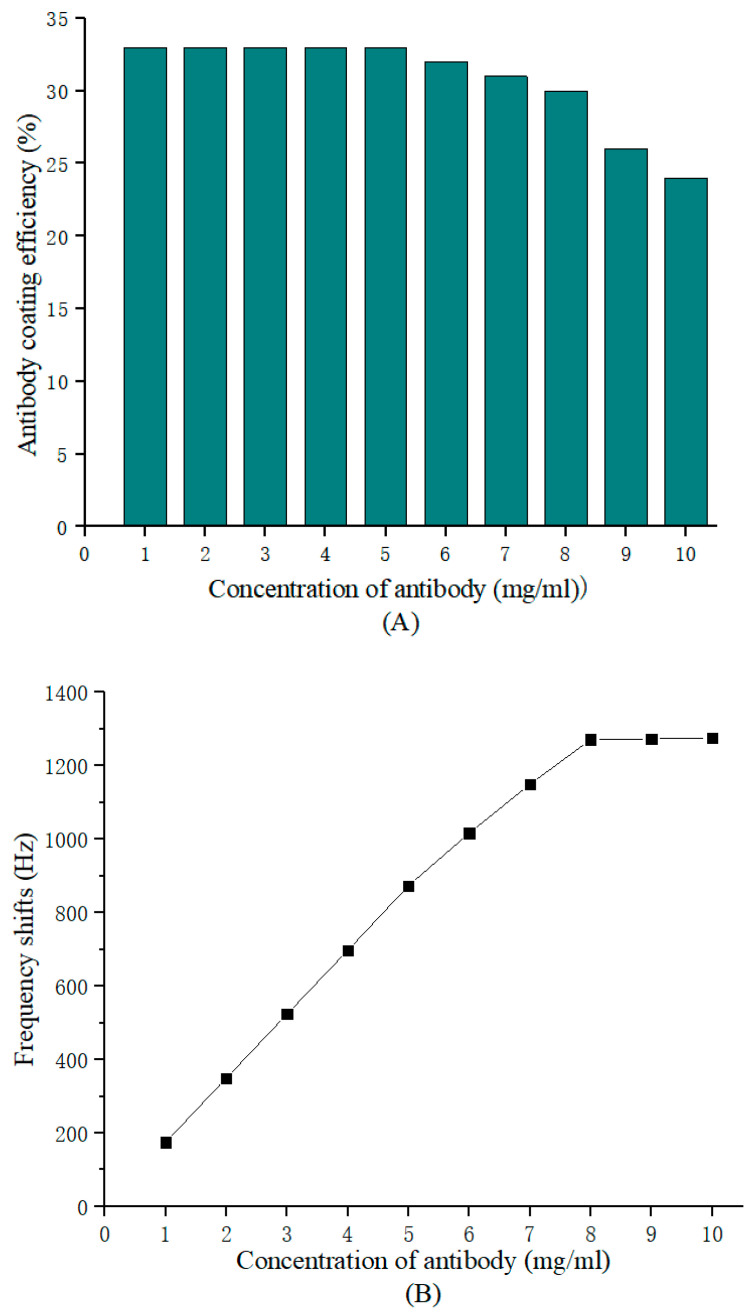
PZ biosensor response for different concentrations of AFP antibody. (**A**) Coating efficiency. (**B**) Frequency variation.

In the application of the PZ-sensing immunoassay, it is most convenient to adopt the standard curve method for quantitative analysis. For a certain antibody–antigen pair, there is an infinite number of standard curves when their concentrations are varied. Then, finding the optimal standard curve is vital in PZ sensor clinical application. First, 2 μL AFP antibody solution of various concentrations (1 mg/mL–10 mg/mL) were deposited on each working crystal to determine the optimal coated antibody concentration. The antibody coating efficiency on the surface of the quartz crystal electrode was calculated using Equation (3), as follows:

(3) $$Antibody\;coating\;efficiency=\frac{\Delta m}{m}\times 100\%$$

where *m* is the total antibody mass loaded on the surface of the quartz crystal gold electrode, and Δm is the antibody mass stably immobilized on the surface of the electrode, which is the mass that causes the frequency change in the resonator in Equation (1). The antibody coating efficiency and sensor frequency response linearly vary with the coated antibody concentration, as shown in Figure 3.

**Figure 4:**
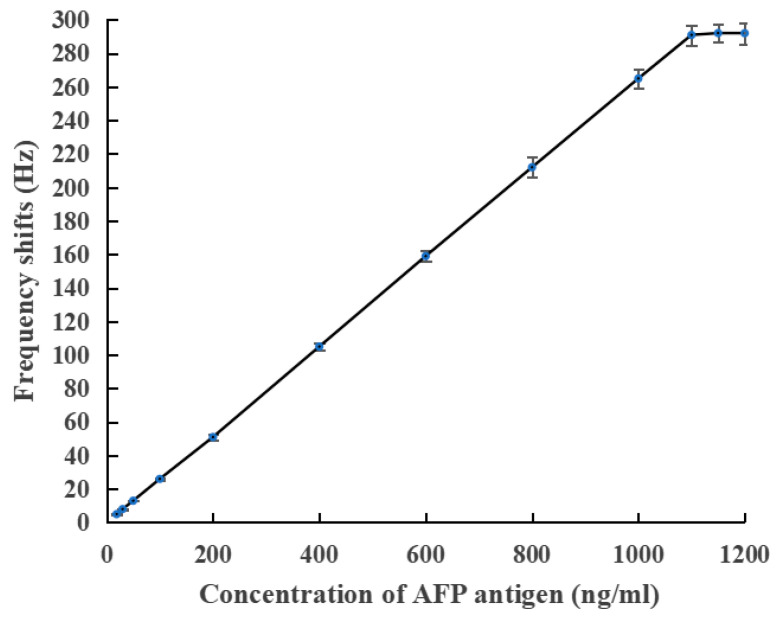
Optimal standard curve for AFP determination using PZ biosensor.

The maximum antibody concentration in the linear region with a coated efficiency above 30% was selected as the optimal immobilized antibody concentration (8 mg/mL). Then, the optimal standard curve, illustrated in Figure 4, was obtained by incubating different concentrations (0–1200 ng/mL) of AFP with 8 mg/mL deposited anti-AFP for 12 min. The frequency change in the coated crystals before and after incubation versus the AFP concentration plots is linear from 18.8 to 1100 ng/mL, with a determination limit of 18.8 ng/mL. Beyond 1100 ng/mL, saturation was encountered on account of the completion binding of all sites of the immobilized antibody. If the concentration of AFP in clinical samples exceeded 1100 ng/ ml, extra detection was needed after the serum was diluted. Let x (in ng/mL) and y (in Hz) stand for the antigen concentration and PZ biosensor frequency response, respectively; then, the fitted equation is y=0.2652x−0.5238, and the coefficient of determination (R^2^) is one. Moreover, the limit of blank (LOB) of this sensing system is 12.5 ng/mL. Since the mass sensitivity of the quartz crystal resonator is affected by the geometry of the electrode on the quartz crystal surface [27], the detection limit can be reduced by reducing the geometry of the upper electrode [28]. Therefore, the PZ biosensor assay designed with a continuous flow and whole-electrode occupation is of great potential in the AFP-level analysis of clinical sera.

### 3.6. Determination AFP Level in Clinical Serum Samples

The frequency response of PZ biosensors consists of the mass loading effect and liquid damping effect [29]. Therefore, in order to eliminate the liquid damping effect and any possible systematic errors, the changed frequencies of the reference crystals must be subtracted from the total frequency shifts of the working crystals. The levels of AFP were determined from the responses of the specific interaction (ΔF) using the standard curve. Ten clinical serum samples (four from an asymptomatic individual who underwent liver cancer screening, four from confirmed and treated liver cancer patients, one from a pregnant women, and one from a lung cancer patient) were then analyzed to determine the AFP level using the PZ sensor. The assay was conducted at 25 °C using a flow rate of 100 μL/min. Meanwhile, a radioimmunoassay (RIA), another established laboratory serum analysis technique, was also employed to analyze these samples. Table 4 describes the results of using the two methods.

As can be seen from Table 4, antibody activity was completely retained by the SPA coating method, with no cross-reactivity with the lung cancer marker, and an abnormal AFP level was found in the liver-cancer-screened serum samples (number 3). At the same time, the relative deviation in the above two detection methods was no more than 3.7%, and the correlation coefficient r was 0.9998, and the data were in good agreement for the two analytical methods. Most notably, 12 min was needed for each analysis cycle, while this lasted 3 h for RIA. At the end of each sample measurement, the crystal could be regenerated with 1.2 M NaOH solution.

### 3.7. Regeneration of PZ Quartz Crystal Biosensor

**Figure 5:**
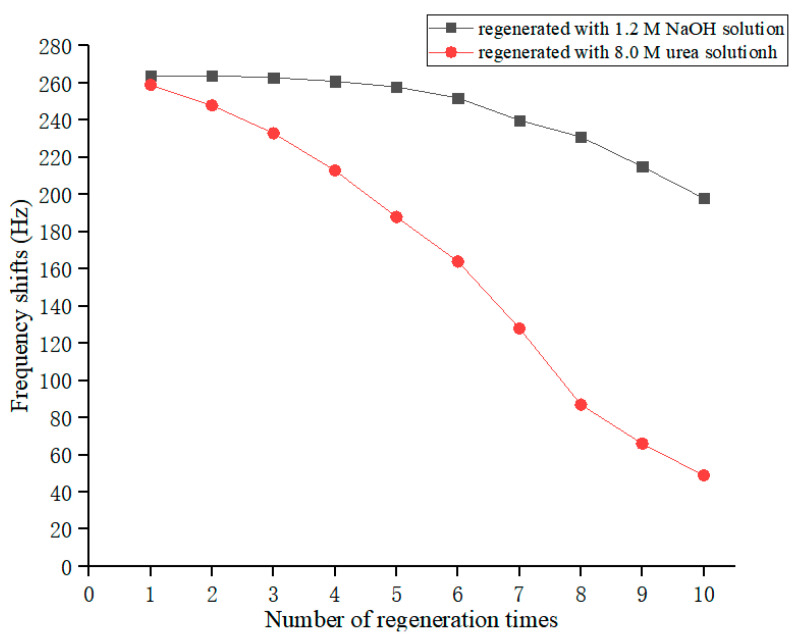
Relationship between the frequency response of PZ biosensor and the number of regeneration times.

Gold electrode regeneration plays an important part in the development of a PZ quartz crystal biosensor and can commercialize it. To make the quartz crystal reusable, the antigen–antibody complex needs to be dissociated with a dissociating agent. In order not to impair the activity of the antibody, the concentration and time of action of the reagent need to be carefully determined. Here, the used quartz crystals were regenerated with a 1.2 M NaOH solution and an 8.0 M urea solution for 2 min, respectively, rinsed with double-distilled water and PBS buffer, and then, used for the detection of standard sera with the same concentration (1000 ng/mL) to detect the regeneration effect. The frequency response is shown in Figure 5.

It was found that the regenerated quartz crystal still had good frequency response characteristics, but its response gradually decreased with the increase in regeneration cycles. Among them, the NaOH solution had a better regeneration ability, and the frequency response marginally changed within five regeneration cycles. After that, the response of the quartz crystal gradually declined, which may be related to the destruction of the antibody-immobilized film. After 10 regeneration cycles, the value of the frequency response dropped to 75% of the original value. Therefore, the regeneration times of quartz crystals should not be too long, so as not to affect the test results.

## 4. Conclusions

A PZ quartz crystal biosensor detection system was designed to analyze the dynamic binding properties of antigen–antibody pairs in tumor markers. Four features of the system can be summarized: only one side of the crystal can be in contact with the serum, there must be a continuously high injection rate, whole-electrode surface occupation must be ensured for the reaction surface, and the quartz crystal must work at its third harmonic frequency, as driven by a harmonic suppression network. The developed PZ-sensing system can reach stable oscillation, improve the mass transport, greatly reduce the depletion layer deposition on the reaction surface, accelerate the antigen–antibody reaction rate, shorten the overall analysis time and be used to perform liver cancer screening. Compared with the existing means of determining alpha-fetoprotein, such as ELISA, CLIA and RIA, the biggest advantage of this immune detection device is that the detection time is shorter and the operation is simpler. A short assay time (only 12 min), a label-free nature, good accuracy and high specificity are properties that make this PZ biosensor system a promising tool for the clinical screening of tumor markers, as well as for novel high-affinity binding drug development. Thus, it is necessary to further examine this immunoassay device to obtain an ideal detection limit in molecular diagnostics.

## Figures and Tables

**Table 1:** Flow liquid measurement (25 °C; 1 mL 500 ng/mL antigen solution at 100 μL/min flow rate).

Monitored Time (Min)	Flow Liquid Measurement			
	Antigen BindingΔf(Hz)	Rate of Antigen Binding (ng/min)	Average Antigen Binding Rate (ng/min)	Completion of Immunoreaction (%)
1	13	48.9	45.9	9.85
2	26	48.9		19.7
3	39	48.9		29.5
4	51	48		38.6
5	63	47.4		47.7
6	75	47		56.8
7	86	46.2		65.2
8	96	45.1		72.7
9	105	43.9		79.5
10	115	43.2		87.1
11	124	42.4		93.9
12	132	41.3		100

**Table 2:** Static liquid measurement (25 °C; 1 mL 500 ng/mL antigen solution).

Monitored Time (Min)	Static Liquid Measurement			
	Antigen Binding Δf (Hz)	Rate of Antigen Binding (ng/min)	Average Antigen Binding Rate (ng/min)	Completion of Immunoreaction (%)
1	13	48.9	38.3	9.85
2	26	48.9		19.7
3	38	47.6		28.8
4	47	44		35.6
5	55	41.4		41.7
6	63	38.9		47.7
7	70	37.6		53
8	75	35.2		56.8
9	78	32.6		59.1
10	80	30		60.6
11	82	28.4		62.1
12	82	25.7		62.1

**Table 3:** Kinetic reaction parameters of AFP antigen–antibody pair for PZ immunosensor measurement.

Sample	Fit Model	$k_{\mathrm{on}}$ (M^−1^s^−1^)	$k_{\mathrm{off}}$ (s^−1^)	K_D_ (M)
Human AFP antigen	1:1 Langmuir binding model	$5.58\times 10^{4}$	$1.79\times 10^{-5}$	$3.21\times 10^{-10}$

**Table 4:** Comparison of AFP levels in real serum sample determination using PZ biosensor and RIA.

Serum Samples	PZ Biosensor		RIA		Relative Deviation (%)	Correlation Coefficient (r)
	AFP Levels (ng/mL)	Analysis Time	AFP Levels (ng/mL)	Analysis Time		
1 ^a^	22.6	12 min	21.8	3 h	3.7	0.9998
2 ^a^	20.7		21.4		−3.3	
3 ^a^	782.2		775.3		0.9	
4 ^a^	24.4		24.9		−2	
5 ^b^	684.5		695.2		−1.5	
6 ^b^	921.4		913.7		0.8	
7 ^b^	842.8		840.1		0.3	
8 ^b^	759.7		747.9		1.6	
9 ^c^	409.9		398.1		3	
10 ^d^	21.8		22.2		−1.8	

^a^ Sera from individuals who underwent liver cancer screening. ^b^ Sera from liver cancer patients. ^c^ Serum from pregnant woman. ^d^ Serum from lung cancer patient.

## Data Availability

The data that support the findings of this study are available from the corresponding author upon request.
